# Assessing Intraoperative Tumor-to-Background Ratios Across Different Subsites of the Oral Cavity Using an Integrin-Specific Fluorescent Tracer

**DOI:** 10.3390/cancers18121910

**Published:** 2026-06-11

**Authors:** Bo E. Zweedijk, Martha F. A. D. Osei-Agyeman, Lorraine J. Lauwerends, Dominic J. Robinson, Hamed Abbasi, Jens F. de Gijsel, Hetty Mast, Brend P. Jonker, José A. U. Hardillo, Dominiek A. Monserez, Aniel Sewnaik, Robert J. Baatenburg de Jong, Cornelis Verhoef, John V. Frangioni, Sjors A. Koppes, Denise E. Hilling, Alexander L. Vahrmeijer, Stijn Keereweer

**Affiliations:** 1 Department of Otorhinolaryngology, Head and Neck Surgery, Erasmus MC Cancer Institute, University Medical Center Rotterdam, 3015 GD Rotterdam, The Netherlands; 2 Department of Surgical Oncology and Gastrointestinal Surgery, Erasmus MC Cancer Institute, University Medical Center Rotterdam, 3015 GD Rotterdam, The Netherlands; 3 Department of Imaging Physics, Delft University of Technology, 2628 CD Delft, The Netherlands; 4 Department of Oral and Maxillofacial Surgery, Erasmus MC Cancer Institute, University Medical Center Rotterdam, 3015 GD Rotterdam, The Netherlands; 5 Curadel Surgical Innovations, 28120 Hunters Ridge Blvd, Suites 6-7, Bonita Springs, FL 34135, USA; 6 Department of Pathology, Erasmus Medical Center, University Medical Center Rotterdam, 3015 GD Rotterdam, The Netherlands; 7 Department of Surgery, Leiden University Medical Center, 2333 ZA Leiden, The Netherlands

**Keywords:** fluorescence imaging, fluorescence-guided surgery, image-guided surgery, molecular imaging, integrin, optical imaging, oral squamous cell carcinoma, oral cancer, head and neck cancer, tumor-specific

## Abstract

**Background/Objectives:** Intraoperative fluorescence imaging (FI) with tumor-targeted tracers offers a promising approach to improve surgical precision in cancer surgery. cRGD-ZW800-1, an integrin-targeted fluorescent tracer, has previously demonstrated safety, tumor specificity, and utility in detecting inadequate margins in oral cancer. During this study, we observed variability in background fluorescence between different subsites of the oral cavity. Therefore, this study aimed to systematically evaluate intraoperative in vivo and ex vivo mucosal contrast ratios across various oral cavity subsites using FI with cRGD-ZW800-1. **Methods:** Thirty-one patients with oral squamous cell carcinoma underwent intraoperative FI following intravenous injection of cRGD-ZW800-1 at least 18 h preoperatively. In vivo imaging was performed using the Quest Spectrum platform. In addition, ex vivo FI of the resected specimen was performed using the Pearl Trilogy Small Animal Imaging System. As these ex vivo images were obtained under uniform and controlled acquisition conditions, they allow for direct comparison with the intraoperative fluorescence signals. Fluorescence intensities and tumor-to-background ratios (TBRs) were assessed per oral subsite using manually drawn regions of interest (ROIs) on the tumor and adjacent healthy mucosa using Quest’s Spectrum Software, version 4.8.2, (in vivo images) and the Pearl’s integrated software ImageStudio version 6.2 (ex vivo images). A TBR ≥ 1.5 was considered sufficient. **Results:** Under uniform imaging settings, all samples exhibited adequate contrast (TBR ≥ 2.3), allowing clear tumor visualization and precise evaluation of mucosal margins on final histopathology. Notably, intraoperative in vivo contrast in the posterior located maxillary alveolar process was comparatively lower, which was attributable to suboptimal imaging conditions and subsite-specific background fluorescence. **Conclusions:** Our findings indicate that, although contrast varies across different oral subsites, all specimens exhibited sufficient ex vivo mucosal contrast to allow reliable tumor delineation. As in vivo imaging may be affected by subsite-specific background fluorescence and inherent limitations of intraoperative imaging geometry, fluorescence signals should be interpreted in conjunction with standard visual and tactile assessment. Due to anatomical constraints, different oral subsites may appear within the same field of view, which can influence perceived signal intensity. Therefore, intraoperative ex vivo fluorescence evaluation is recommended for signal interpretation.

## Simple Summary

Surgical removal is the primary treatment for oral cancer, and clear margins are essential for good outcomes. Fluorescence imaging (FI) using the integrin-targeted near-infrared dye cRGD-ZW800-1 is safe, tumor-specific, and helps detect inadequate margins during surgery. Because background fluorescence varies across oral cavity subsites, we systematically assessed intraoperative in vivo and ex vivo mucosal contrast ratios. Under standardized imaging conditions, all specimens showed sufficient contrast (tumor-to-background ratio ≥ 2.3), enabling reliable tumor delineation and accurate mucosal margin assessment on final histopathology. Lower in vivo contrast was observed in the posterior maxillary alveolar process, likely due to less optimal imaging conditions (greater camera distance and acute angle) combined with subsite-specific physiological background fluorescence. Overall, FI with cRGD-ZW800-1 effectively differentiates tumor tissue from healthy mucosa across oral cavity subsites. These subsite-specific contrast insights can improve surgical precision and support more accurate tumor resections.

## 1. Introduction

Oral squamous cell carcinoma (OSCC) is an important global health problem accounting for approximately 390,000 new cases and 189,000 deaths each year [1]. Surgical removal remains the cornerstone of oral cancer treatment, during which the surgeon aims to achieve clear surgical margins. Although prognosis in OSCC is influenced by multiple histopathological, biological, and patient-related factors, including depth of invasion, perineural invasion, worst pattern of invasion (WPOI), and comorbidity, most of these factors cannot be influenced intraoperatively by the surgeon. In contrast, surgical margin status, which is strongly associated with local recurrence, the need for adjuvant therapy, and poorer survival outcomes, represents one of the few prognostic factors that can be directly optimized during surgery [2,3,4,5,6,7,8,9]. Therefore, techniques that support accurate intraoperative margin assessment, such as fluorescence-guided surgery, are of particular clinical interest.

Enhanced intraoperative visualization of oral cancer is expected to improve the percentage of complete tumor resections [2]. Fluorescence imaging (FI) supports this by enabling real-time visualization of tumor tissue using a systemically administered, tumor-targeted, tracer that emits near-infrared (NIR) light [10]. This signal is captured with NIR camera systems, providing surgeons with real-time visual contrast between tumor and healthy tissue. Fluorescence-guided surgery originated with metabolic tracers such as 5-aminolevulinic acid (5-ALA), which enabled intraoperative tumor visualization in neurosurgical oncology and established the clinical feasibility of fluorescence-guided resection [11,12]. For accurate intraoperative interpretation of fluorescence signals, reliable contrast assessment requires not only substantial tracer accumulation within the tumor relative to the surrounding tissue, but also on the requirement that imaging conditions are equivalent for both regions; parameters such as camera distance and camera angle must be kept consistent to ensure accurate and reproducible contrast assessment. This ultimately assists in achieving complete tumor resections in oral cancer.

In our recently completed clinical trial, we evaluated the efficacy of FI using cRGD-ZW800-1, an integrin-targeted fluorescent tracer, for the detection of inadequate resection margins during oral cancer surgery. This technique demonstrated robust in vivo fluorescent contrast as early as two hours post-administration. Additionally, ex vivo intraoperative FI successfully identified all inadequate margins [13]. Notably, in vivo FI revealed fluorescent signal emission from several healthy oral cavity sites, such as the gingiva, attributable to the physiological expression of integrins [14,15]. A related study by de Wit et al. evaluated the EGFR-targeted fluorescent tracer cetuximab-800CW in oral cancer and demonstrated tumor-specific tracer uptake [16]. However, fluorescence background across oral cavity subsites was not assessed. It is plausible that physiological expression of molecular targets may vary between anatomical regions, potentially affecting tumor-to-background contrast. Considering that effective mucosal delineation using FI requires sufficient contrast, it is critical to determine how this physiological integrin expression impacts the practical application of this imaging technique in vivo. In this context, where histopathological confirmation is not available, reliance on accurate contrast is essential for real-time decision making.

Therefore, the primary objective of this subsequent study is to assess the in vivo fluorescent mucosal contrast ratios across various anatomical subsites of the oral cavity with FI using cRGD-ZW800-1, and to compare these in vivo measurements with ex vivo contrast, where imaging conditions are standardized. Furthermore, we aim to evaluate whether this tracer can reliably differentiate tumor tissue from adjacent healthy mucosa in each individual subsite of the oral cavity.

## 2. Materials and Methods

### 2.1. Study Design

This study is part of a clinical trial for resection margin evaluation in patients with OSCC using integrin-targeted FI [13]. This study was approved by the local Ethics Review Committee (METC Erasmus MC; MEC-2020-0149) and conducted in full compliance with the principles of the Declaration of Helsinki of 1975, the ICH GCP guidelines, and the laws and regulations of the Netherlands. The study is registered in the European Clinical Trials Database (EudraCT 2019-003416-30) and ClinicalTrials.gov (NCT04191460).

Thirty-one patients with biopsy-proven OSCC who underwent surgical resection at the Erasmus MC Cancer Institute in Rotterdam were included. All patients provided written informed consent prior to participation in the study. Detailed descriptions of the study design, patient population, investigational agent, imaging protocols, and primary outcome measures have been reported elsewhere [13]. During this study, the optimal dosing and administration-to-imaging time interval of the fluorescent tracer were evaluated, primarily based on in vivo multi-diameter single-fiber reflectance single-fiber fluorescence (MDSFR/SFF) spectroscopy. This technique calculates the intrinsic fluorescence of cRGD-ZW800-1 by correcting the raw fluorescence spectrum for tissue optical properties (e.g., autofluorescence and absorption), to account for variations in tissue composition and ensure that any observed increase in signal is truly due to higher tracer concentration [17,18]. Open surgical-field FI of the tumor and surrounding healthy tissue was performed using the CE-marked Quest Spectrum Platform (Olympus Corporation, Tokyo, Japan). First, the tumor was delineated mucosally using the parallel tagging method without the use of FI, aiming for a 10 mm mucosal margin [19]. FI was then performed, and any changes to the surgical plan based on fluorescence were documented. In addition to these intraoperative in vivo measurements, ex vivo FI of the resected specimen was performed using the Pearl Trilogy Small Animal Imaging System (LI-COR Biosciences Inc., Lincoln, NE, USA). As these ex vivo images were obtained under uniform and controlled acquisition conditions, they allow for direct comparison with the intraoperative fluorescence signals and histopathological findings.

### 2.2. Data Analysis

**Figure 1:**
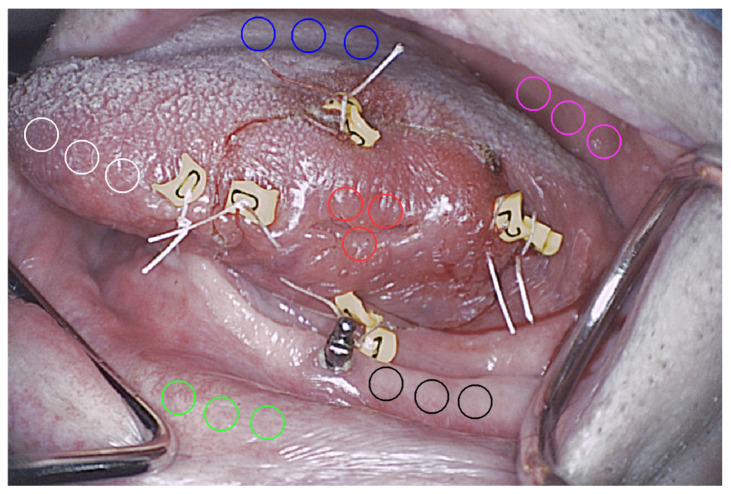
Region-of-interest (ROI) placement in oral cavity of a representative patient, made with Quest Spectrum Software (TBR tool v1.3). Dorsal tongue (blue), gingiva (black), lateral tongue (white), palatum durum (pink), labial mucosa (green), and tumor (red).

**Figure 2:**
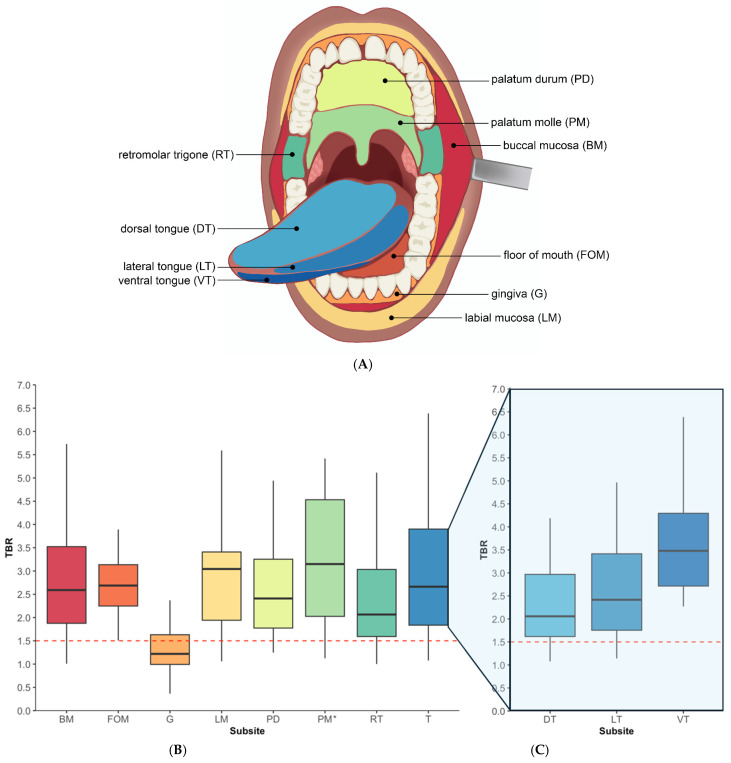
Schematic overview of all color-coded anatomical sites within the oral cavity (**A**), with corresponding TBR measurements across all subsites (**B**) and sub-group analysis for specific tongue surfaces (**C**). The horizontal dashed line represents the TBR threshold of 1.5 [20]. *: Although the palatum molle is officially part of the oropharynx, we included this subsite here as it may represent surrounding healthy tissue in the context of oral cavity tumors. TBR: tumor-to-background ratio, BM: buccal mucosa, FOM: floor of mouth, G: gingiva, LM: labial mucosa, PD: palatum durum, PM: palatum molle, RT: retromolar trigone, T: tongue, DT: dorsal tongue, LT: lateral tongue, VT: ventral tongue.

For contrast evaluation, in vivo images acquired prior to tumor resection were used. Regions of interest (ROI) were manually drawn spanning the mucosal tumor, and others in healthy surrounding tissue (background) using Quest’s Spectrum Software (TBR tool v1.3). Figure 1 illustrates an example of ROI placement in a patient with a lateral tongue tumor. Surrounding tissue included all visible subsites of the oral cavity as defined in Figure 2A. The quotient of the mean fluorescence intensity (MFI) of the tumor and visible surrounding subsites constituted the in vivo tumor-to-background ratio (TBR) (Figure 2B,C). Each patient could have multiple background regions, resulting in multiple TBR calculations per patient. Additionally, individual contrast ratios were determined based on the fluorescence intensity of the tumor compared to the directly adjacent oral subsite (background). The number and size of ROIs (for both tumor and background) were kept consistent within each patient to ensure reliable TBR calculations. For ex vivo contrast evaluation, ROIs were drawn in both the tumor and the directly adjacent mucosal subsite (background) using the Pearl’s integrated software (ImageStudio version 6.2; LI-COR Biosciences Inc., Lincoln, NE, USA). In line with existing literature, a TBR ≥ 1.5 was deemed adequate [20].

For statistical analysis, continuous variables were summarized using descriptive statistics (n, mean/median, SD/interquartile range (IQR)). Categorical variables were expressed as count and percentages. Predetermined subgroup analysis of anatomical subsites was performed. A *p*-value of <0.05 was considered statistically significant. Statistical analysis of the data was done using R software (version 4.3.2).

## 3. Results

We reviewed 31 patients that had been operated between July 2022 and April 2025. Within this cohort were six women and 23 men, with a mean age at the time of surgery of 66 years (SD = 12.8). All patients had tumors showing similar histological phenotypes. The vast majority had well- or moderately differentiated, keratinizing, OSCC (n = 29), the remaining two patient had poorly differentiated tumors, both exhibiting focal keratinization. Table 1 presents a summary of patient characteristics.

### 3.1. In Vivo Tumor-to-Background Ratio Analysis Across Anatomical Subsites

Considering the variability in TBR across the different subsites, non-parametric statistical analyses were conducted, resulting in the calculation of median TBR values (see Table 2). All anatomical subsites demonstrated adequate in vivo contrast (TBR > 2.0), with the exception of the gingiva. The highest contrast was observed in the ventral tongue (median TBR 3.48, IQR 2.70–4.39), whereas the gingiva demonstrated the lowest TBR (median TBR 1.22, IQR 0.99–1.63). This lower gingival contrast was mainly driven by a single patient with a tumor located at the maxillary alveolar process, who demonstrated a comparatively low in vivo TBR of 1.07 when analysed at the individual patient level using the directly adjacent oral subsite as background (Appendix A, Table A1). Apart from this patient, all remaining patients demonstrated sufficient in vivo contrast at the individual patient level. Figure 2 provides an illustrative depiction of each anatomical subsite in the oral cavity and its corresponding TBR.

### 3.2. Ex Vivo Tumor-to-Background Ratio Analysis Across Anatomical Subsites

To further contextualize the observed variation in in vivo mucosal contrast, ex vivo mucosal contrast ratios were determined for all patients under rigorously standardized conditions, including fixed sample-to-detector distance and perpendicular illumination, representing optimal imaging geometry. These ex vivo measurements provided a controlled framework for interpreting fluorescence signals in relation to histopathological findings and subsite-specific contrast differences observed intraoperatively.

**Figure A1:**
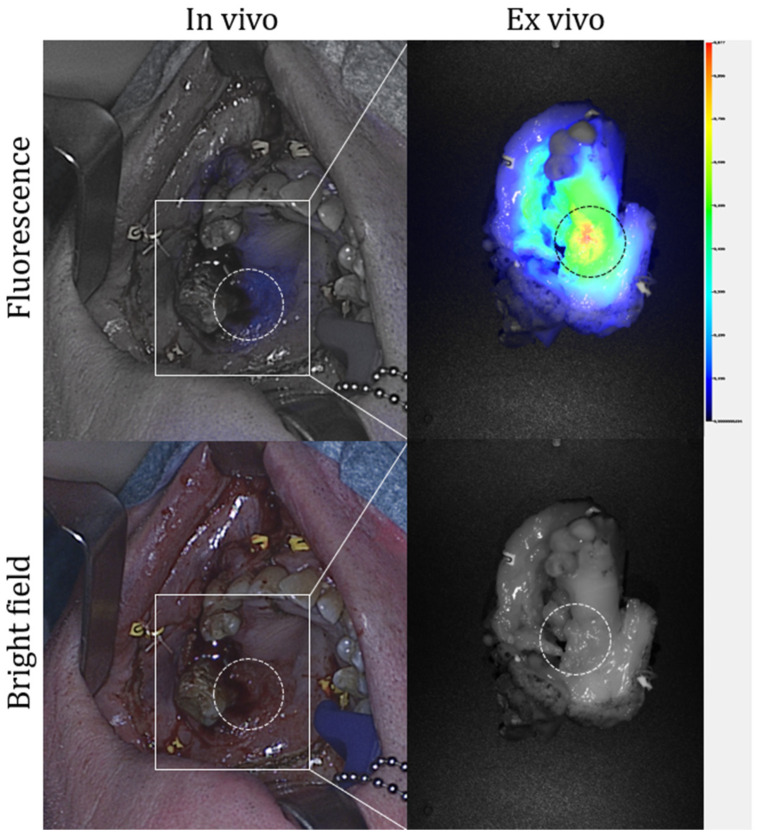
In vivo and ex vivo back-table fluorescence of a tumor located at the right alveolar process of the maxilla. In vivo fluorescence imaging demonstrates signals from both the tumor and surrounding tissue, resulting in reduced contrast. Ex vivo imaging of the resected specimen provides a clearer distinction, with the tumor exhibiting markedly higher fluorescence intensity compared to adjacent healthy tissue. This indicates that the reduced contrast in vivo is primarily due to suboptimal imaging conditions. The resection is outlined in the quadrangle, in which the tumor is outlined by a dashed circle.

Under these controlled conditions, ex vivo FI demonstrated a high mucosal contrast, with a median ex vivo TBR of 3.91 (IQR 3.00–5.57). Even at the individual patient level, all tumors exhibited adequate ex vivo contrast, with no ex vivo TBR below 2.3 (Appendix A, Table A1). Notably, the one patient who showed insufficient in vivo contrast demonstrated a markedly high ex vivo TBR of 2.57, indicating that the low intraoperative contrast was not due to insufficient tracer accumulation, but rather to suboptimal camera access and exposure during surgery. Figure A1 (Appendix A) provides both the in vivo and ex vivo fluorescence images of a maxillary alveolar process tumor, revealing sufficient contrast ex vivo.

Additionally, in vivo FI were compared to spectroscopy-based measurements, to validate whether the observed signal differences are attributable to actual variation in tracer concentration. This analysis confirmed sufficient contrast in nearly all patients (Appendix A, Table A1). Notably, the patient with the tumor located in the alveolar process, who exhibited relatively low in vivo contrast, demonstrated adequate contrast according to both ex vivo and spectroscopy-based fluorescence findings (patient 15). One patient with a tumor located in the floor of mouth exhibited a comparatively low spectroscopy-based TBR (patient 6, TBR 1.17), which was discordant with the corresponding in vivo and ex vivo TBR measurements. This discrepancy is likely attributable to the background measurement being obtained from the contralateral floor of mouth, including the sublingual gland. Our previous clinical trial demonstrated physiological integrin expression in salivary gland tissue, resulting in elevated fluorescence signals [13]. Collectively, these findings suggest that the observed discrepancy reflects site-specific physiological tracer uptake rather than inadequate tumor contrast or methodological limitations of the imaging approach.

### 3.3. cRGD-ZW800-1 Uptake in Non-Tumor Tissues

**Figure 3:**
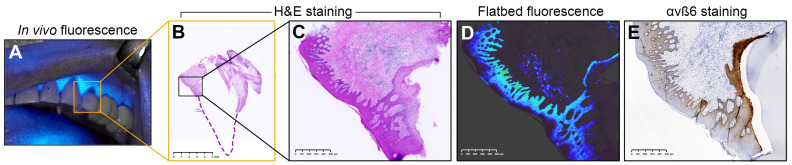
Fluorescence in the gingiva. In vivo fluorescence was consistently observed in the gingiva (**A**). H&E-stained sections demonstrate the overall tissue morphology and epithelial architecture (**B**,**C**). Flatbed fluorescence imaging identified a localized signal at the epithelial junction (**D**), corresponding to strong αvβ6 expression on immunohistochemistry (**E**). Reproduced from Zweedijk et al., [13] Near-infrared fluorescence imaging-guided surgery using cRGD-ZW800 to improve surgical resection margins in oral cancer: a phase I/II feasibility trial, Nature Communications, 2026, licensed under CC BY 4.0.

**Figure A2:**
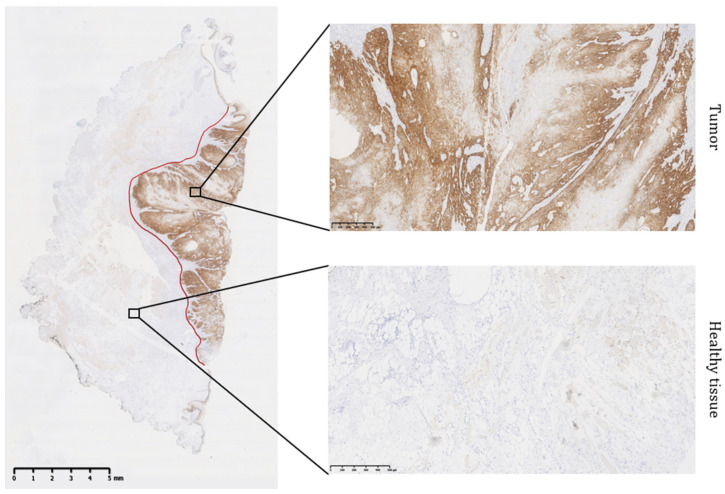
Integrin (αvβ6) expression of a lateral tongue tumor. The tumor is delineated in red.

Throughout the study, a consistent fluorescence signal was observed in clinically tumor-negative gingival tissue; In vivo MDSFR/SFF spectroscopy measurements confirmed that the intrinsic fluorescence of gingiva was not significantly higher than that of non-gingival oral subsites (0.021 vs. 0.020, *p* = 0.81), indicating that this higher signal was not caused by autofluorescence. This suggests that the increased fluorescence signal in gingival tissue is more likely attributable to increased integrin expression and/or non-specific tracer uptake rather than intrinsic autofluorescence. To assess the biological basis of this signal, additional ex vivo FI was performed on selected microscopy slides containing gingiva using a flatbed scanner (Odyssey M or Odyssey CLx, LI-COR Biosciences Inc., Lincoln, NE, USA). In parallel, immunohistochemistry (IHC) staining for αvβ6 integrin expression was conducted on 4 µm formalin-fixed, paraffin-embedded (FFPE) sections of healthy gingiva. The results were compared with corresponding hematoxylin and eosin (H&E) stained slides to assess the spatial correlation between fluorescence signal and αvβ6 expression. This revealed a high expression of the integrin αvβ6 in the gingiva, making the high fluorescence signal in this region highly specific (Figure 3). Furthermore, elevated integrin expression in oral tumors was confirmed by comparing healthy and tumorous lateral tongue epithelium, demonstrating absent to minimal αvβ6 expression in normal tongue tissue and pronounced αvβ6 expression in the adjacent tumor (Appendix A, Figure A2).

## 4. Discussion

This clinical study systemically evaluates intraoperative mucosal contrast ratios across various anatomical subsites in the oral cavity using FI with cRGD-ZW800-1. Our findings demonstrated adequate mucosal contrast across all oral cavity subsites. Tumors located at the maxillary alveolar process demonstrated a relatively low in vivo contrast, which was attributable to suboptimal imaging conditions related to the posterior anatomical location. In these cases, FI was performed at acute angles that precluded perpendicular camera alignment with the tumor surface, while adjacent mucosa was positioned more proximal to the camera. Collectively, these factors contributed to reduced intraoperative contrast, underscoring the well-established influence of camera geometry and tissue topology on fluorescence measurements [21,22]. Following surgical excision, ex vivo imaging was performed under standardized imaging conditions, revealing uniformly high mucosal contrast across all oral cavity subsites, including posteriorly located lesions (TBR ≥ 2.3). These findings indicate that the reduced in vivo contrast was not the result of insufficient tracer accumulation but rather reflected limited camera access and exposure during surgery. In addition, in vivo spectroscopy measurements demonstrated that the intrinsic fluorescence of gingival tissue was not significantly higher than that of non-gingival oral subsites, indicating that the elevated fluorescence signals observed in gingival tissue during in vivo imaging were not driven by autofluorescence. Instead, these signals are consistent with increased tracer concentrations, in line with the elevated integrin expression demonstrated in the present study and previously reported literature [13,14,15].

Our findings underscore that posteriorly located lesions pose technical challenges related to camera access and positioning, an important consideration when interpreting in vivo fluorescence signals in these specific anatomical contexts. Accordingly, maximal effort should be directed toward optimizing camera positioning and visualization during in vivo imaging, while intraoperative ex vivo specimen-based imaging can be considered as a complementary approach when in vivo assessment is technically limited. Importantly, despite the in vivo imaging challenges encountered in specific anatomical subsites, clinically relevant fluorescence signals remained detectable in deeper or less accessible regions. This was exemplified by a case involving a deeply situated maxillary sinus tumor, in which residual fluorescence signal was clearly detectable in the wound bed and corresponded with a positive fresh frozen section analysis, demonstrating that intraoperative FI can assist margin assessment despite suboptimal camera alignment.

In addition to in vivo FI, we evaluated spectroscopy-based TBR measurements to provide a quantitative assessment of tracer uptake while correcting for tissue optical properties. Although this approach offers a more precise estimation of true tracer concentration, its clinical relevance is limited, as sufficient contrast was already consistently observed using the standard intraoperative imaging systems without optical property correction. From a clinical perspective, the ability to reliably visualize contrast using the intraoperative imaging devices is paramount, as this reflects real-time intraoperative conditions and workflow. Consequently, while spectroscopy-based analysis serves as a valuable validation tool, it does not appear to be essential for routine clinical implementation when adequate contrast is readily achieved with standard FI.

Our findings revealed subsite-specific variability in mucosal contrast ratios across the oral cavity. As previously noted, gingival subsites, including the alveolar processes, demonstrated comparatively lower contrast in vivo (median TBR 1.22, IQR 0.99–1.63). In addition, tumors located at the retromolar trigone exhibited lower TBR values in vivo relative to other subsites (median TBR 2.07, IQR 1.40–3.73). Similarly to the alveolar process, the retromolar trigone constitutes a posterior anatomical subsite where intraoperative imaging conditions are frequently constrained by limited camera access, non-perpendicular imaging angles, and close proximity of adjacent mucosa, factors that may adversely affect fluorescence contrast. Furthermore, tumors of the dorsal tongue were found to have comparatively lower contrast (median TBR 2.06, IQR 1.60–3.09). Unlike posterior subsites, this finding is less likely attributable to restricted camera access and may instead reflect intrinsic tissue characteristics, such as epithelial thickness, keratinization, and local vascularization. Notably, previous literature has identified the dorsal tongue as one of the oral subsites with the greatest epithelial thickness [23], a factor that may influence fluorescence signal behaviour through increased light absorption and scattering [21]. Collectively, these observations underscore that both anatomical accessibility and subsite-specific tissue properties contribute to variability in intraoperative fluorescence contrast and should be considered when interpreting fluorescence imaging results.

To our knowledge, intraoperative fluorescence contrast ratios of various subsites have not yet been reported using this approach. Existing studies on fluorescence-guided surgery predominantly report ex vivo or histologically validated contrast values, and only a limited number of publications have explored in vivo fluorescence contrast for oral cancer [16,24,25]. These studies, however, do not differentiate between specific oral subsites and therefore provide little insight into potential regional variability. Such variability is plausible, as tracer uptake and fluorescence signal intensity are known to differ across (oral) subsites due to inherent biological differences in tissue composition and optical properties. By performing all measurements in vivo at multiple intraoral locations, the present study is the first to demonstrate the potential applicability of this FI approach across different anatomical regions within the oral cavity.

Our study provides valuable and novel insights into the intraoperative fluorescence contrast ratios specific to cRGD-ZW800-1, offering a detailed and nuanced understanding of how this tracer performs across various oral cavity subsites. By systematically assessing these contrast ratios in vivo and ex vivo, we establish important reference points that guide the interpretation of fluorescence signals during surgery. This improved understanding helps surgeons distinguish tumor tissue from surrounding healthy mucosa with greater accuracy, thereby enhancing the precision of tumor resection. Ultimately, these findings reinforce the clinical utility of cRGD-ZW800-1 as a reliable imaging agent, supporting its role in facilitating more informed surgical decision-making in oral cancer surgery.

## 5. Conclusions

This clinical study demonstrates that FI using cRGD-ZW800-1 effectively distinguishes tumor tissue from healthy mucosa across all oral cavity subsites during oral cancer surgery. Subsite-specific variability in intraoperative fluorescence contrast was observed, reflecting the combined influence of anatomical accessibility and intrinsic tissue characteristics. In particular, posteriorly located lesions pose technical challenges during in vivo imaging, especially with regard to camera access and positioning, which may affect fluorescence signal interpretation. By providing detailed insights into the intraoperative fluorescence contrast ratios specific to cRGD-ZW800-1, our findings offer a nuanced understanding that has the potential to improve surgical precision and support more accurate tumor resections.

## Figures and Tables

**Table 1:** Patient characteristics.

	n (%)
Age, years (mean, SD)	66 (12.8)
Gender	
Female	8 (25.8)
Male	23 (74.2)
Tumor site	
Alveolar process of the mandibula	2 (6.5)
Alveolar process of the maxilla	3 (9.7)
Buccal mucosa	1 (3.2)
Floor of mouth	6 (19.4)
Lateral tongue	15 (48.4)
Posterior tongue	1 (3.2)
Retromolar trigone	2 (6.5)
Ventral tongue	1 (3.2)
Tumor stage	
T1	5 (16.1)
T2	17 (54.8)
T3	5 (16.1)
T4	4 (12.9)
cRGD-ZW800-1 dose (mg/kg)	
0.01	3 (9.7)
0.025	21 (67.7)
0.05	7 (22.6)

**Table 2:** Overview of the median in vivo TBR of each healthy anatomical subsites in the oral cavity (background) compared to the tumor. As each patient could have multiple background regions, the total number of TBR measurements per anatomical subsite (n) exceeded the total number of patients.

Healthy Background	n	Median In Vivo TBR (Q1–Q3)
Buccal mucosa	21	2.59 (1.88–3.52)
Floor of mouth	14	2.69 (2.18–3.15)
Gingiva	21	1.22 (0.99–1.63)
Labial mucosa	23	3.04 (1.94–3.41)
Palatum durum	12	2.41 (1.76–3.54)
Palatum molle	6	3.15 (1.79–4.86)
Retromolar trigone	4	2.07 (1.40–3.73)
Tongue	53	2.66 (1.84–3.90)
Dorsal tongue	20	2.06 (1.60–3.09)
Lateral tongue	21	2.42 (1.75–3.42)
Ventral tongue	12	3.48 (2.70–4.39)

## Data Availability

Non-identifiable imaging, safety, clinical, and laboratory data are available from the corresponding author upon reasonable request (S. Keereweer, s.keereweer@erasmusmc.nl). In accordance with Dutch regulations, the data will be stored for a minimum period of 15 years.

## Abbreviations

The following abbreviations are used in this manuscript:

FFPE	Formalin-Fixed Paraffin-Embedded
FI	Fluorescence Imaging
H&E	Hematoxylin and Eosin
ICH GCP	International Council for Harmonization–Good Clinical Practice
IHC	Immunohistochemistry
IQR	Interquartile Range
MDSFR/SFF	Multi-Diameter Single-Fiber Reflectance/Single-Fiber Fluorescence
MFI	Mean Fluorescence Intensity
NIR	Near-Infrared
OSCC	Oral Squamous Cell Carcinoma
ROI	Region of Interest
SD	Standard Deviation
TBR	Tumor-to-Background Ratio

## Appendix A

**Table A1:** In vivo and ex vivo mucosal contrast of subsites directly adjacent to the tumor for each individual patient.

#	Tumor Location	In Vivo TBR	Ex Vivo TBR	In Vivo Spectroscopy-Based TBR
1	Lateral tongue	2.21	N/A ^a^	2.07
2	Lateral tongue	1.67	6.78	2.20
3	Posterior tongue	2.15	5.43	2.08
4	Retromolar trigone	2.54	2.81	2.45
5	Lateral tongue	2.09	5.71	2.81
6	Floor of mouth	1.86	2.39	1.17 *
7	Lateral tongue	2.49	2.48	3.84
8	Lateral tongue	4.19	3.34	1.67
9	Lateral tongue	4.53	9.24	1.80
10	Floor of mouth	3.12	3.32	21.23
11	Alveolar process of the mandibula	1.94	3.24	2.84
12	Retromolar trigone	1.68	3.07	2.94
13	Alveolar process of the mandibula	1.51	N/A ^b^	2.12
14	Alveolar process of the maxilla	1.07 **	2.57	2.61
15	Floor of mouth	2.65	3.95	4.73
16	Lateral tongue	1.71	2.53	3.59
17	Floor of mouth	N/A ^c^	3.43	2.27
18	Alveolar process of the maxilla	2.17	3.87	2.36
19	Alveolar process of the maxilla	3.35	4.40	8.87
20	Lateral tongue	2.66	2.80	2.79
21	Lateral tongue	3.05	7.11	2.50
22	Ventral tongue	4.97	4.67	2.24
23	Lateral tongue	2.86	3.23	6.19
24	Lateral tongue	2.69	2.67	2.06
25	Lateral tongue	2.23	4.68	9.73
26	Lateral tongue	5.69	7.85	4.67
27	Buccal mucosa	1.91	5.37	3.28
28	Lateral tongue	3.34	7.18	2.63
29	Floor of mouth	3.90	NA ^b^	2.42
30	Floor of mouth	4.14	5.52	5.58
31	Lateral tongue	5.81	9.69	2.61

N/A: not available; TBR: tumor-to-background ratio. * In this patient, the low spectroscopy-based TBR likely resulted from background measurements including the contralateral sublingual gland. ** In this patient, comparatively low in vivo TBR was caused by suboptimal intraoperative camera positioning. ^a^ Ex vivo TBR analysis was not feasible because appropriate closed-field images were unavailable. ^b^ Closed-field FI could not be performed due to a malfunction of the imaging device. ^c^ In vivo TBR analysis was not feasible due to lack of images capturing the directly adjacent background.
